# COVID-19 Pandemic Development in Jordan—Short-Term and Long-Term Forecasting

**DOI:** 10.3390/vaccines9070728

**Published:** 2021-07-02

**Authors:** Tareq Hussein, Mahmoud H. Hammad, Pak Lun Fung, Marwan Al-Kloub, Issam Odeh, Martha A. Zaidan, Darren Wraith

**Affiliations:** 1 Department of Physics, The University of Jordan, Amman 11942, Jordan; mhm0179430@ju.edu.jo; 2 Institute for Atmospheric and Earth System Research (INAR/Physics), University of Helsinki, FI-00014 Helsinki, Finland; pak.fung@helsinki.fi (P.L.F.); martha.zaidan@helsinki.fi (M.A.Z.); 3 Department of Physics, Prince Faisal Technical College, Amman 11134, Jordan; pftcmdr@rjaf.mil.jo; 4 Department of Basic Sciences, Al Zaytoonah University of Jordan, Amman 11733, Jordan; odehi.m@zuj.edu.jo; 5 Joint International Research Laboratory of Atmospheric and Earth System Sciences, School of Atmospheric Sciences, Nanjing University, Nanjing 210023, China; 6 School of Public Health and Social Work, Queensland University of Technology, Brisbane 4000, Australia; d.wraith@qut.edu.au

**Keywords:** linear forecast, white-box model, vaccination, public immunity

## Abstract

In this study, we proposed three simple approaches to forecast COVID-19 reported cases in a Middle Eastern society (Jordan). The first approach was a short-term forecast (STF) model based on a linear forecast model using the previous days as a learning data-base for forecasting. The second approach was a long-term forecast (LTF) model based on a mathematical formula that best described the current pandemic situation in Jordan. Both approaches can be seen as complementary: the STF can cope with sudden daily changes in the pandemic whereas the LTF can be utilized to predict the upcoming waves’ occurrence and strength. As such, the third approach was a hybrid forecast (HF) model merging both the STF and the LTF models. The HF was shown to be an efficient forecast model with excellent accuracy. It is evident that the decision to enforce the curfew at an early stage followed by the planned lockdown has been effective in eliminating a serious wave in April 2020. Vaccination has been effective in combating COVID-19 by reducing infection rates. Based on the forecasting results, there is some possibility that Jordan may face a third wave of the pandemic during the Summer of 2021.

## 1. Introduction

Vaccination against severe acute respiratory syndrome coronavirus 2 (SARS-CoV-2) is strongly recommended to combat novel coronavirus disease (COVID-19). In the absence of vaccination, other public health measures have been used such as social distancing and the avoidance of crowded places in order to reduce the transmission of COVID-19. When COVID-19 infection rates have shown high increasing trends, public health authorities have enforced lockdowns and curfews.

In order to understand the effect of curfew, lockdown, and vaccination on the COVID-19 pandemic curve, several modelling approaches to forecast the COVID-19 reported cases have been utilized. As Artificial Intelligence (AI) and Machine Learning (ML) applications have been used in various areas [1,2], they have been also used for predictions during the SARS-CoV-2 outbreak. They have also shown effectiveness in improving diagnostic and prognostic processes of COVID-19. Limitations of these methods relate to the quality of reporting, lack of understanding/reporting of social and clinical factors, and slow development of spatial risk maps which have affected prediction accuracy [3,4,5,6,7,8]. Recently, an important factor to be included in prediction is the development of vaccination strategies [9].

The main purpose of these predictive models is to optimize protection and prevent the spread of COVID-19. As such, several strategies have been implemented: (1) identification of suspicious events, (2) large-scale screening, (3) tracking, (4) associations with experimental treatments, (5) pneumonia screening, (6) data and knowledge collection and integration, (7) resource distribution, (8) robotics for medical quarantine, (9) forecasts, and (10) modeling (e.g., Susceptible–Exposed–Infectious–Recovery (SEIR), stochastic transmission models, etc.) and simulation [10,11,12,13,14,15]. In practice, governments use these tools to develop public health measures (e.g., social distancing, lockdown, and closure of facilities, etc.) to contain the spread of the virus.

In the literature, there have been four studies focused on modeling the COVID-19 pandemic outbreak in Jordan [16,17,18,19]. However, these studies focused on the early stage of the COVID-19 pandemic outbreak before the first wave was triggered. While the suggested methods were efficient in predicting the COVID-19 outbreak, they were limited to the curfew period. Therefore, the suggested models were not tested for the complete picture of the COVID-19 pandemic development and evolution over a long-term period. Furthermore, the applied models did not include the scenario of vaccination on limiting the COVID-19 infection rates in Jordan. These models were also quite complicated and difficult to interpret as a forecast for the COVID-19 pandemic in Jordan. Hadid et al. [16] used fractional Chebyshev polynomials, based on the Atangana–Baleanu fractional operator, to describe the growth rate of the COVID-19 infection. They showed that their method provided flexibility and accuracy of introducing approximate solutions. Hadid et al. [16] recommended the use of the same calculus (ABC) to generalize different polynomials to obtain an optimal solution. Kumar et al. [17] used a Hermite wavelets basis to solve the COVID-19 model with time-arbitrary Caputo derivative. At the same time, Saidan et al. [18] mathematically estimated the probable outbreak size of COVID-19 clusters (such as religious, wedding, and industrial activity) using a simple model that can predict the number of COVID-19 cases as a function of time. They adapted the cluster behavior in different countries between February and April 2020 and applied to the Jordan case. Saidan et al. [18] claimed that their model can offer a contact-tracing task with the predicted number of cases, which would help in epidemiological investigations by knowing when to stop. Kheirallah et al. [19] utilized an epidemic model, which was based on a modified susceptible, exposed, infected, and recovered. Their model simulation and trajectories of the COVID-19 pandemic curve in Jordan between February and May confirmed that strict non-pharmaceutical interventions measures seemed to be effective in controlling the COVID-19 epidemic and reducing the reproduction rate. Early strict intervention measures showed evidence of containing and suppressing the disease.

This paper presents three simple approaches to forecast the COVID-19 pandemic spread in a Middle Eastern society (in Jordan) over a long-term period that covered different social conditions such as normal life situations, curfew, lockdown, and vaccination. Relatively simple models are useful in situations where detailed information about the pandemic are not available and/or rely upon quite strong assumptions (e.g., transmission behaviour and vaccination rates). These types of models are also potentially more generalizable to other countries in similar circumstances.

## 2. Materials and Methods

### 2.1. COVID-19 Data

**Figure 1:**
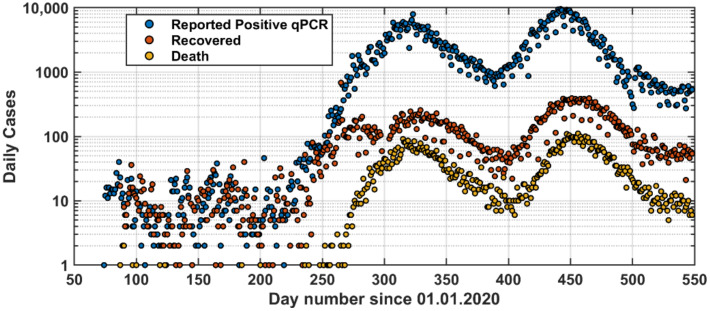
Daily reported cases of positive qPCR tests, recovered, and death since the first case was reported in Jordan (14 March 2020).

**Figure 2:**
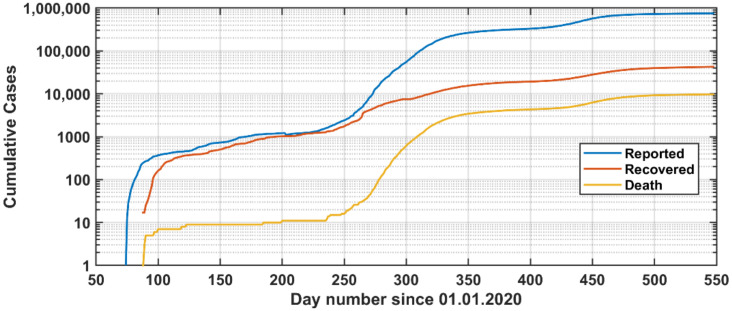
Cumulative reported cases of positive qPCR tests, recovered, and death since the first case was reported in Jordan (14 March 2020).

The COVID-19 database included the daily reported positive cases, recovered cases, and deaths (Figure 1; see also File S1). The source of this data was from official reports of the Ministry of Health in Jordan. The data were collected on a daily basis. Figure 2 shows the corresponding cumulative numbers. The Ministry of Health reports the number of daily qPCR tests and the corresponding positive cases (Figure 1).

**Figure 3:**
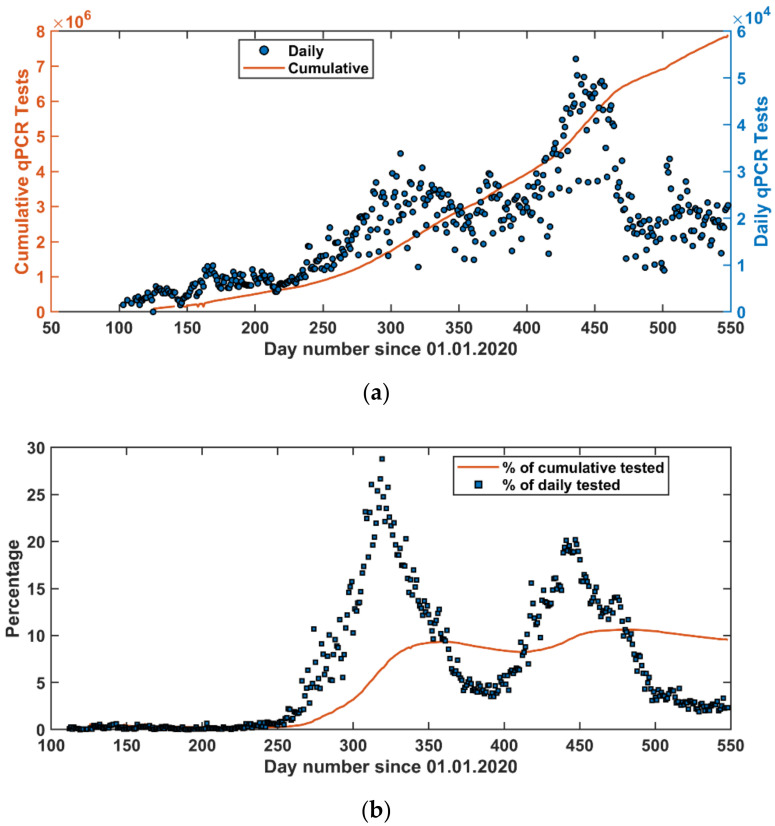
(**a**) Number of daily and cumulative qPCR tests performed in Jordan since the first case was reported (14 March 2020). (**b**) Daily and cumulative percentage of the positive qPCR cases out of the performed tests.

**Figure 4:**
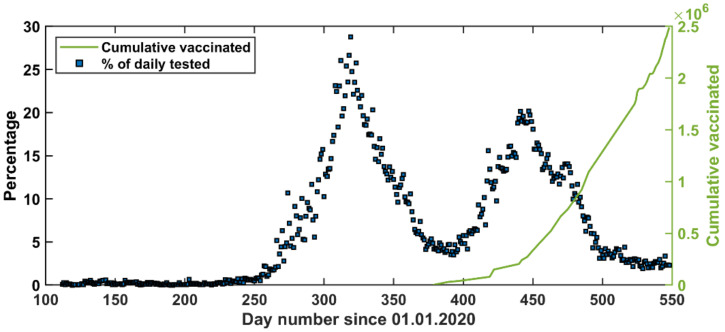
Daily percentage of the positive qPCR cases out of the performed tests and cumulative vaccination (at least once).

While the community has followed the number of reported positive cases, an important number is the percentage of positive cases out of the total number of daily qPCR tests (Figure 3). In fact, assuming random testing, the percentage of positive cases out of the total number of daily qPCR tests is the most important parameter in describing the pandemic situation. Therefore, this is the parameter we considered for our modeling approaches. In January 2021, the COVID-19 vaccination strategy started in Jordan (Figure 4).

### 2.2. Forecast Models for the Postive qPCR Tests

#### 2.2.1. Short-Term Forecast (STF) Model

**Figure 5:**
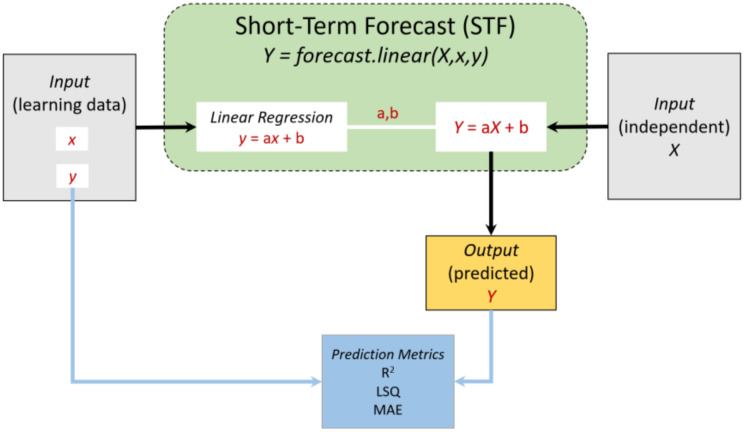
A scheme showing the short-term forecast (STF) model.

The short-term forecast (STF) model is based on a simple linear forecast model (built-in function “*forecast.linear*” in Microsoft Excel, Microsoft office 365, Figure 5)

*Y* = *forecast.linear*(*X*,*x*,*y*) (1)

where *Y* is the predicted percentage of positive qPCR test(s) on day(s) *X*. The learning data are the time range (*x*) and corresponding observed (i.e., reported) positive qPCR tests (*y*). The range of *x* and *y* spans over the previous days (between 5 and 40 days); i.e., the dataset (*x*,*y*) is the learning data for the linear prediction of *Y* on day *X*. The Microsoft Excel *forecast.linear*(*X*,*x*,*y*) function predicts a value based on existing values along a linear trend.

#### 2.2.2. Long-Term Forecast (LTF) Model

**Figure 6:**
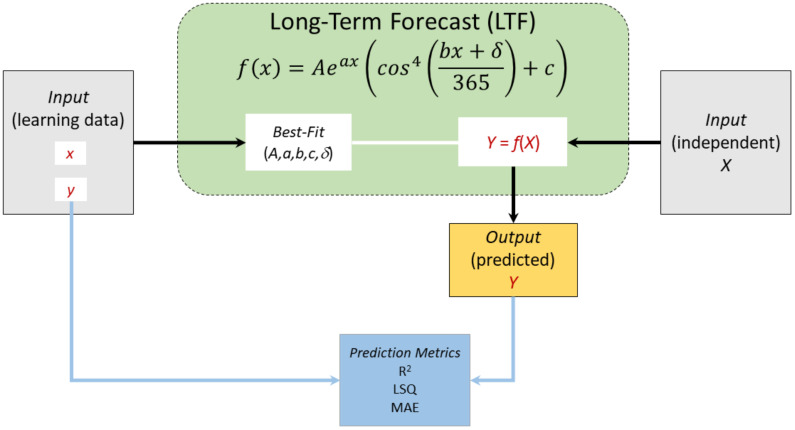
A scheme showing the long-term forecast (LTF) model.

The long-term forecast (LTF) model is based on a white-box approach by choosing the best mathematical function that describes the previously reported percentage of positive daily qPCR tests (as percentage out of the total daily qPCR tests); Figure 6. A preliminary and exploratory analysis for this database indicated that the best fit has the following mathematical form

(2) $$Y=Ae^{-aX}\left(cos^{4}\left(\frac{bX+\delta }{365}\right)+c\right)$$

where *Y* is the predicted percentage of positive qPCR tests on day *X* (as day number after 01.01.2020). The parameters *A*, *a*, *b*, *δ*, and *c* are the average model parameters that best fit this function with the database and are presented in Figure 9. These were 58, 0.003, 9.01, 530, and 0.22, respectively. This function was chosen in analogy with the concepts of some physical phenomena (e.g., damping oscillator) after a slight modification to the power of the trigonometric function.

#### 2.2.3. Hybrid Forecast (HF) Model

**Figure 7:**
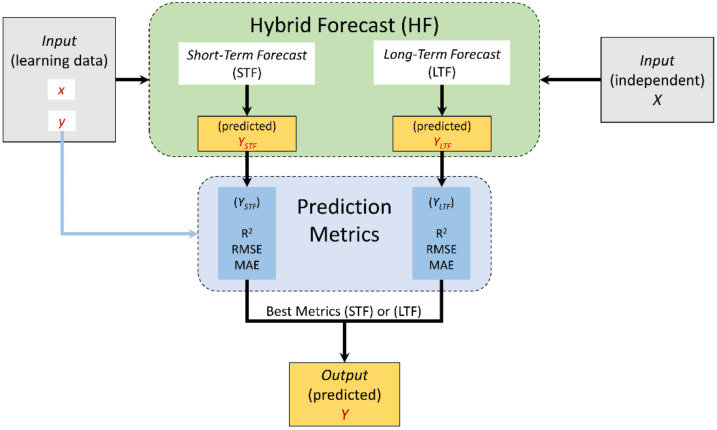
A scheme showing the hybrid forecast (HF) model.

As per the results discussed later in this manuscript, a hybrid forecast (HF) model is suggested to be a combination between the STF and the LTF models (Figure 7) based on the following criteria:

- During curfew and lockdown periods, the HF is applied.
- During normal life condition periods, the LTF model is applied.

In practice, the turning point between the HF and the LTF models was during the first week of October 2020. The HF model can be also extended and applied for the period after the end of the second wave so that the prediction can cope with the effect of vaccination.

Here, it is important to distinguish between “lockdown” and “curfew”. During an emergency-like situation, a “lockdown” is a shutdown forced (under the Disaster Management Act) for public services (i.e., transport, business establishments, educational institutions, restaurants, malls, etc.) which are temporarily closed until the situation improves. A “curfew” is a strict order by the National Center for Security and Crisis Management forcing people to remain at home. Violation of the curfew or the lockdown will have legal consequences such jail or penalty.

#### 2.2.4. Prediction Metrics

The model accuracy was tested by the coefficient of determination (*R*^2^), least-square value (*RMSE*) and mean absolute error (*MAE*), as follows:

(3) $$R^{2}=1-\frac{\sum_{i=1}^{n}{\left({\hat{y}}_{i}-y_{i}\right)}^{2}}{\sum_{i=1}^{n}{\left(y_{i}-\bar{y}\right)}^{2}},$$

(4) $$RMSE=\sqrt{\frac{\sum_{i=1}^{n}{\left({\hat{y}}_{i}-y_{i}\right)}^{2}}{n}},$$

(5) $$MAE=\frac{\sum_{i=1}^{n}\left|{\hat{y}}_{i}-y_{i}\right|}{n},$$

where $y_{i}$ and ${\hat{y}}_{i}$ represent, respectively, the *i*th observed value and the *i*th predicted value. $\bar{y}$ denotes the mean of the observed dataset of n data points. *R*^2^ illustrates the linear association between the observed variable and the predicted output variable by the selected model. The higher the value of *R*^2^, the higher the variability of the predicted variable the model can explain. While both *RMSE* and *MAE* measure the average difference between the observed and the predicted variable, the difference between these two terms lies in that *RMSE* represents the quadratic mean of these differences, yet *MAE* calculates the absolute difference.

## 3. Results and Discussion

### 3.1. Overall Description of the COVID-19 Pandemic in Jordan

The pandemic development is shown in Figure 1 and Figure 2. On 14 March 2020, the first COVID-19 case was reported in Jordan. On 18 March 2020, the government took a series of immediate actions to limit the spread of the COVID-19 pandemic in the whole country.

The curfew was started gradually right after the first case was reported. By the end of April 2020, the curfew was lifted gradually but all ports (boarders, airports, etc.) remained closed until early September 2020; however, only nationals were allowed to return according to scheduled flights and arrivals and had to stay in quarantine for two weeks. After September 2020, daily life returned gradually to normal with partial lockdown during the weekend. Consequently, the first wave of the outbreak started in late September 2020 reaching its maximum in the middle of November 2020 and was over by the middle of January 2021.

A second wave of the pandemic started in early February 2021 reaching its maximum around the middle of March 2021 and was over in middle May 2021. Interestingly, the pandemic waves spanned over a period of approximately three months with about 6 weeks separation (Figure 1 and Figure 3b). Based on daily reported number of COVID-19 cases, the second wave seemed to be stronger than the first peak (Figure 1); however, it was milder than the first wave when looking at the percentage of positive daily qPCR tests (Figure 3b).

### 3.2. Short-Term Forecast (STF)

**Figure 8:**
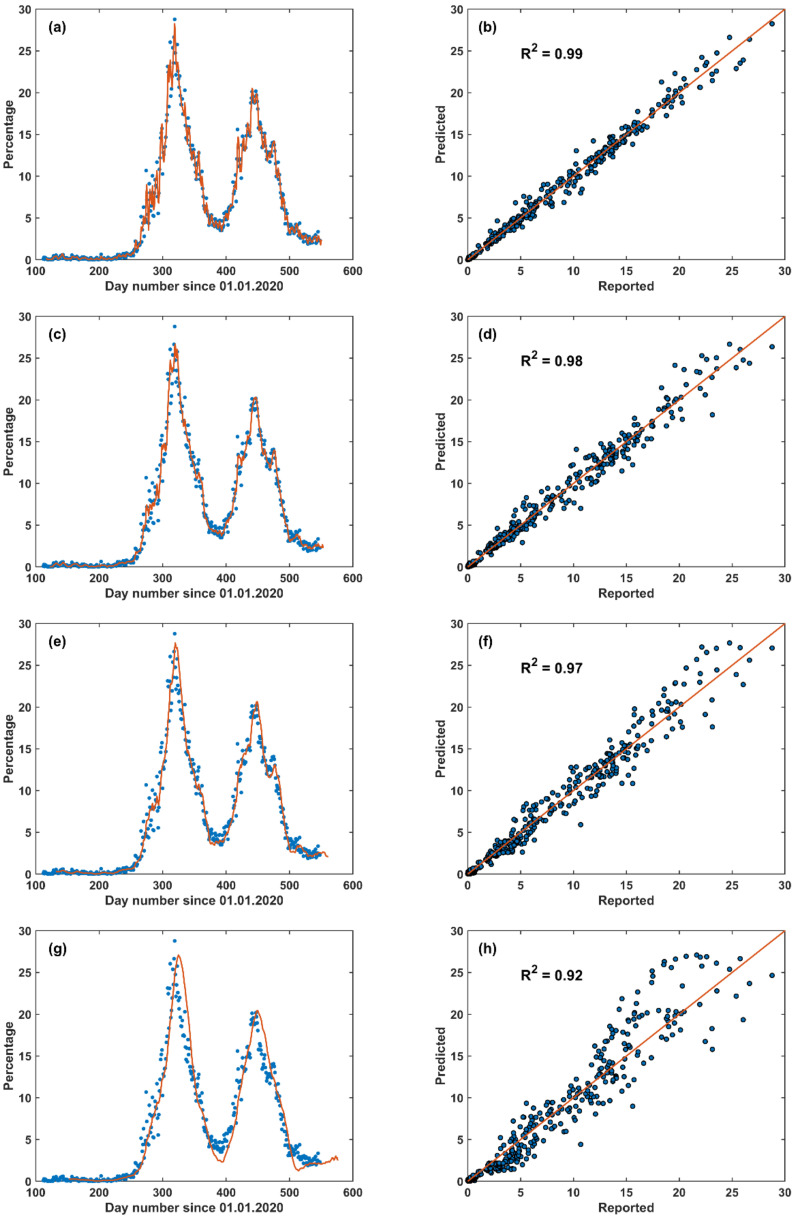
Short-term forecast (STF) models (Equation (1)) for the COVID-19 pandemic in Jordan based on the positive qPCR cases out of the performed daily tests (as percentage out of the total qPCR daily tests): (**a**,**b**) previous 5 days learning, (**c**,**d**) previous 10 days learning, (**e**,**f**) previous 20 days learning, (**g**,**h**) previous 40 days learning. The left panel is the time-series of the percentage of the daily positive qPCR tests percentage and the right panel is the corresponding scatter plots between the forecasted and the reported daily positive qPCR tests percentage. Legend of the left panel: (dots) reported and (line) forecasted.

The STF model was applied by choosing a range of learning previous days: 5, 10, 15, 20, 25, 30, 35, and 40 (Figure 8). The STF model results showed a fast response to the daily changes of the reported data and *R*^2^ estimates are provided in Table 1.

The greater the number of learning days, the lower the *R*^2^ value. However, choosing the five days learning approach is not realistic and almost replicates the reported data and choosing 40 days provides a lag in the forecasted data. Therefore, a reasonable learning period can be 10–20 days prior to the forecasted day.

### 3.3. Long-Term Forecast (LTF)

**Figure 9:**
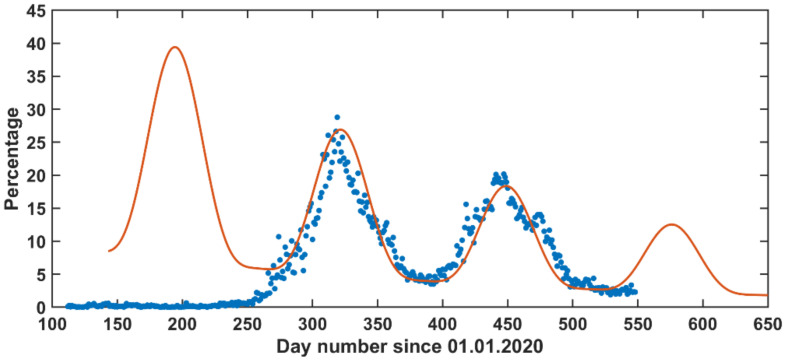
Long-term forecast (LTF) model (Equation (2)) for the COVID-19 pandemic in Jordan based on the positive qPCR cases out of the performed daily tests (as percentage out of the total qPCR daily tests). Legend: (dots) reported and (line) forecasted.

The long-term forecast (LTF) model result is shown in (Figure 9). It looks interesting how the selected function is capable of forecasting the reported COVID-19 cases (first and second reported waves) over a long-term period.

This function suggests a zeroth wave, which was not reported. This zeroth wave (which was supposed to start in April 2020) was not observed because the whole country entered the curfew followed by a strict lockdown. Once the lockdown was lifted gradually, the pandemic waves were reported. Another possible reason for not observing the zeroth peak can be due to limitations in qPCR testing in the beginning of the pandemic; over time, the medical facilities have developed a better infrastructure with respect to qPCR testing and hospital admissions. Excluding the zeroth wave, the LTF prediction metrics were *R*^2^ = 0.84, RMSE = 2.81, and MAE = 2.16.

The third wave is expected to start in June and end in early September 2021. Noticeably, if the zeroth wave occurred, it would be stronger than the first reported wave. Interestingly, the upcoming wave is expected to be milder than the first and the second wave. The waves are appearing milder and milder possibly due to social immunity and vaccination (Figure 4).

### 3.4. Hybrid Forecast (HF)

**Figure 10:**
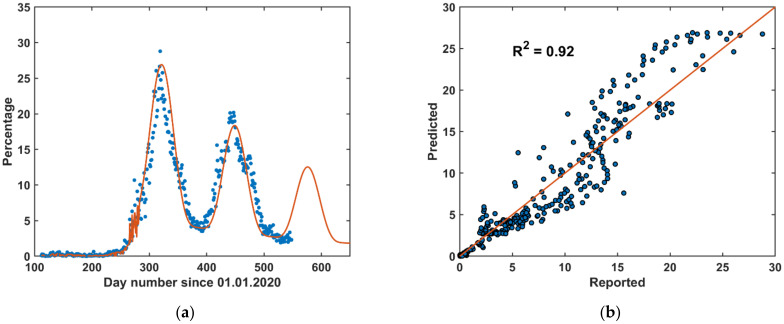
Hybrid forecast (HF) model: (**a**) is the time-series of the pandemic and (**b**) is the scatter plot between the predicted and the reported percentage of the daily positive qPCR daily testes.

The hybrid forecast (HF) model result is shown in (Figure 10). It is plausible that the HF is capable of forecasting the reported COVID-19 cases at any time regardless of the lockdown, curfew, or normal life conditions. The HF is superior to the LTF because the LTF is not capable of forecasting the COVID-19 cases during the curfew period (before June 2020). The HF is also superior to the STF because the STF is not capable of accurately forecasting the COVID-19 cases for more than 5 days. The HF model prediction metrics were *R*^2^ = 0.92, RMSE = 2.15, and MAE = 1.35.

According to the HF forecast, a third peak is expected to start in July 2021. However, vaccination rates might have a significant effect on reducing the impact of this peak. This is speculated by observing the percentage curve (Figure 3 and Figure 4), which shows that the percentage was leveling around 4% between the first and the second peak and after the second peak the percentage has decreased steadily reaching values around 2%. As such, a slight modification to the HF model can be introduced in the future to include the effect of vaccination by reintroducing the STF approach with a specific number of days as training data.

### 3.5. Discussion

Each approach has its own advantages and disadvantages. The short-term approach strongly predicts the upcoming five days and copes with the daily sudden changes of the COVID-19 pandemic. Although the long-term approach lacks these advantages, it is performing well as a forecasting model for the COVID-19 pandemic for the upcoming months. However, the long-term approach presented in this study may fail, for example, when social activity changes or the authorities define new actions to limit the pandemic outbreak. Therefore, a hybrid approach combining the short-term and the long-term approaches is an advantageous solution to predict sudden changes as well as the long-term situation in the pandemic outbreak (Figure 10). The forecasting models presented here were based on simple approaches. Nevertheless, they were capable of predicting the pandemic perfectly as complementary methods to each other.

The modelling approach has some limitations including that the qPCR testing is randomly undertaken throughout the community. This may ignore clustering effects, although there isn’t strong evidence that testing was only isolated to particular areas. The models also do not include (explicitly) accurate information about the vaccination trend, although they are able to model the observed (empirical) effect of vaccination on the outbreak. In the current situation where vaccination rates are low, this may be a more practical approach.

Looking closely at the reported COVID-19 pandemic in Jordan (Figure 1), the reported positive qPCR tests were higher in the second wave than those in the first wave, but both waves were prolonged over a period of about three months. This gives an indication that the second wave was stronger than the first one. However, looking at the ratio of positive qPCR tests to the total daily tests reveals that the second wave was weaker than the first one (Figure 4). Three reasons can explain this. First, people who became infected in the first wave are not likely to be infected in the second wave, which was within six months of the first wave. Second, vaccination was started in early January 2021 in Jordan; and this enhances the immunity of the public. Following these two lines of reasoning, the third wave is expected to be in August 2021 (based on the long-term forecast) but will be even weaker than the second wave (Figure 10a). Third, the community has become more aware of social distancing after each wave.

Overall and for the Jordan case, the forecast modelling revealed the effect of lockdown and curfew in postponing the pandemic waves. In fact, the medical infrastructure development had an advantage from this postponement to accommodate and adapt before the outbreak hits a large fraction of the Jordanian society. The vaccination trend was another factor in limiting the infection rate within the Jordanian society as clearly seen from the waves’ outbreaks, which were decreasing in values but remained within a similar time span. Therefore, other countries can learn from the Jordanian case in controlling the COVID-19 outbreaks and protecting their societies by following an adaptive lockdown/curfew orders and effectively vaccinating the community as fast as possible.

## 4. Conclusions

The COVID-19 pandemic in Jordan started late and developed slowly in the beginning of 2020. This was due to careful and immediate interventions applied by the Jordanian government. The main advantage of such strategic actions postponed the pandemic waves until the medical infrastructure became ready to cover the medical needs for the infected cases. However, once the COVID-19 pandemic outbreak started in late 2020, the pandemic waves escalated with a length of three months and four months separation (measured between the peaks’ maxima). The second wave was weaker than the first wave as a result of social immunity and vaccination, which started in the beginning of 2021.

Here, three simple prediction approaches were proposed to forecast the COVID-19 reported cases in a Middle Eastern society (Jordan):

- The first approach, which was a short-term forecast (STF) model, was based on a linear forecast model with the previous ten days as a learning data-base for the upcoming five days forecasting. This short-term approach can cope with sudden daily changes in the pandemic.
- The second approach, which was a long-term forecasting (LTF) model, was based on a mathematical formula that best describes the up-to-date pandemic situations in Jordan. This long-term approach can be utilized to predict the upcoming waves’ occurrence and strength.
- A hybrid approach merging both the STF and the LTF models is the best choice to predict the pandemic.

According to the LRF model results, a zeroth wave, which was not reported, was to start in April 2020. This zeroth wave was not observed because the whole country entered the curfew followed by a strict lockdown. Once the lockdown was lifted gradually, the pandemic waves were reported. Another possible reason for not observing the zeroth peak can be due to limitations in the infrastructure of the medical facilities with respect to the capacity of qPCR testing and hospital admissions at the beginning of the COVID-19 pandemic.

A third wave is expected to start in June 2021 and end in early September 2021. The next wave appears milder than the previous one because of social immunity and vaccination.

The accuracy of these approaches was seen as excellent. The STF model with 15 learning days had metrics as follows: *R*^2^ = 0.98, RMSE = 1.09, and MAE = 0.69. The LTF model (excluding the zeroth wave) had *R*^2^ = 0.84, RMSE = 2.81, and MAE = 2.16.

The outcomes of this research can be extended to societies with similar COVID-19 spread and outbreaks. The prediction of COVID-19 can help understand the pandemic spread and outbreak in a society.

## Figures and Tables

**Table 1:** A table showing the evaluation metrics of short-term forecast (STF), long-term forecast (LTF) and hybrid forecast (HF) model.

Model	Number of Learning Days	*R* ^2^	*RMSE*	*MAE*
STF	5	0.990	0.68	0.41
	10	0.981	0.95	0.58
	15	0.975	1.09	0.69
	20	0.969	1.24	0.79
	25	0.958	1.46	0.92
	30	0.947	1.68	1.06
	35	0.934	1.89	1.20
	40	0.921	2.12	1.35
LTF	first wave onwards	0.844	2.81	2.16
HF		0.919	2.15	1.35

## Supplementary Materials

The COVID-19 data used in this manuscript and the forecast modelling are available online at https://www.mdpi.com/article/10.3390/vaccines9070728/s1, File S1: Data sheet and figures.
